# Feed-forward neural networks using cerebral MR spectroscopy and DTI might predict neurodevelopmental outcome in preterm neonates

**DOI:** 10.1007/s00330-020-07053-8

**Published:** 2020-07-18

**Authors:** T. Janjic, S. Pereverzyev, M. Hammerl, V. Neubauer, H. Lerchner, V. Wallner, R. Steiger, U. Kiechl-Kohlendorfer, M. Zimmermann, A. Buchheim, A. E. Grams, E. R. Gizewski

**Affiliations:** 1 Department of Neuroradiology, Medical University of Innsbruck, Anichstraße 35, 6020 Innsbruck, Austria; 2 Neuroimaging Research Core Facility, Medical University of Innsbruck, Innsbruck, Austria; 3 Department of Paediatrics II, Neonatology, Medical University of Innsbruck, Innsbruck, Austria; 4 Institute of Psychology, University of Innsbruck, Innsbruck, Austria

**Keywords:** Premature infants, Neurodevelopmental disorders/diagnosis, Magnetic resonance spectroscopy, Diffusion tensor imaging, Neural networks, computer

## Abstract

**Objectives:**

We aimed to evaluate the ability of feed-forward neural networks (fNNs) to predict the neurodevelopmental outcome (NDO) of very preterm neonates (VPIs) at 12 months corrected age by using biomarkers of cerebral MR proton spectroscopy (^1^H-MRS) and diffusion tensor imaging (DTI) at term-equivalent age (TEA).

**Methods:**

In this prospective study, 300 VPIs born before 32 gestational weeks received an MRI scan at TEA between September 2013 and December 2017. Due to missing or poor-quality spectroscopy data and missing neurodevelopmental tests, 173 VPIs were excluded. Data sets consisting of 103 and 115 VPIs were considered for prediction of motor and cognitive developmental delay, respectively. Five metabolite ratios and two DTI characteristics in six different areas of the brain were evaluated. A feature selection algorithm was developed for receiving a subset of characteristics prevalent for the VPIs with a developmental delay. Finally, the predictors were constructed employing multiple fNNs and fourfold cross-validation.

**Results:**

By employing the constructed fNN predictors, we were able to predict cognitive delays of VPIs with 85.7% sensitivity, 100% specificity, 100% positive predictive value (PPV) and 99.1% negative predictive value (NPV). For the prediction of motor delay, we achieved a sensitivity of 76.9%, a specificity of 98.9%, a PPV of 90.9% and an NPV of 96.7%.

**Conclusion:**

FNNs might be able to predict motor and cognitive development of VPIs at 12 months corrected age when employing biomarkers of cerebral ^1^H-MRS and DTI quantified at TEA.

**Key Points:**

*• A feed-forward neuronal network is a promising tool for outcome prediction in premature infants.*

*• Cerebral proton magnetic resonance spectroscopy and diffusion tensor imaging can be used for the construction of early prognostic biomarkers.*

*• Premature infants that would most benefit from early intervention services can be spotted at the time of optimal neuroplasticity.*

**Electronic supplementary material:**

The online version of this article (10.1007/s00330-020-07053-8) contains supplementary material, which is available to authorized users.

## Introduction

Very preterm infants (VPIs) are at increased risk of cerebral injury [1–3]. White matter injury (WMI) is the foremost cause of chronic neurological impairment in surviving VPIs, predominantly manifested in the form of cerebral palsy and cognitive and learning disabilities [4]. As the extent of the WMI visualised on conventional MRI does not always correlate with the neurodevelopmental outcome (NDO) [5, 6], a more precise assessment with advanced quantitative techniques is indispensable in order to recognise the VPIs at risk at the time of optimal neuroplasticity and provide them with early supportive intervention services.

Quantification of DTI-based metrics such as fractional anisotropy (FA) and mean diffusivity (MD) provides the microstructural characterisation and integrity of white matter [7–9]. FA is characterised by the degree of diffusion anisotropy and is higher the greater the organisation and myelination of the white matter [10]. In contrast, MD represents the average water diffusion [11] and is high in unmyelinated tissue with high water content [12, 13]. Proton spectroscopy (^1^H-MRS), on the other hand, reflects the metabolic composition of the brain tissue, which changes in developing brain due to the maturation and myelination processes [14], but also in cases of structural damage [15]. Therefore, the abnormalities in white matter microstructure and metabolism reflected by DTI and ^1^H-MRS in VPIs may indicate delays in maturation processes or reveal subtle WMI, not obvious on conventional MRI [16]. As a result, employing DTI and ^1^H-MRS may improve the overall prognostic accuracy for developmental outcomes [17, 18], assessed mostly by Bayley Scales of Infant and Toddler Development, a standardised tool for evaluating motor and cognitive function [19].

However, the prediction of NDO remains challenging due to a complex, prematurity-related WMI pathomechanism and a large number of confounders influencing its evolvement. Recently, neural networks (NNs), a segment of artificial intelligence, have opened up new horizons for identifying sophisticated data causalities. This approach is used to extract patterns and detect trends that are too complex to be noticed by either humans or other computer techniques [20, 21]. An artificial neural network with one hidden layer, called “feedforward neural network” (fNN), can approximate arbitrarily well any continuous function of several real variables and is widely used for various regression and classification tasks [22].

We aimed to evaluate the ability of fNNs to predict the NDO of VPIs at 12 months corrected age by using cerebral ^1^H-MRS and DTI biomarkers at term-equivalent age (TEA).

## Methods

### Study participants

This prospective study was approved by the ethics committee of the Medical University of Innsbruck (AN 2014-0131 336/4.13).

All eligible neonates born before 32 gestational weeks were invited for an MRI scan at TEA as part of the follow-up routine programme for VPIs of the Department of Neonatology, Innsbruck Medical University Hospital, between September 2013 and December 2017.

**Fig. 1:**
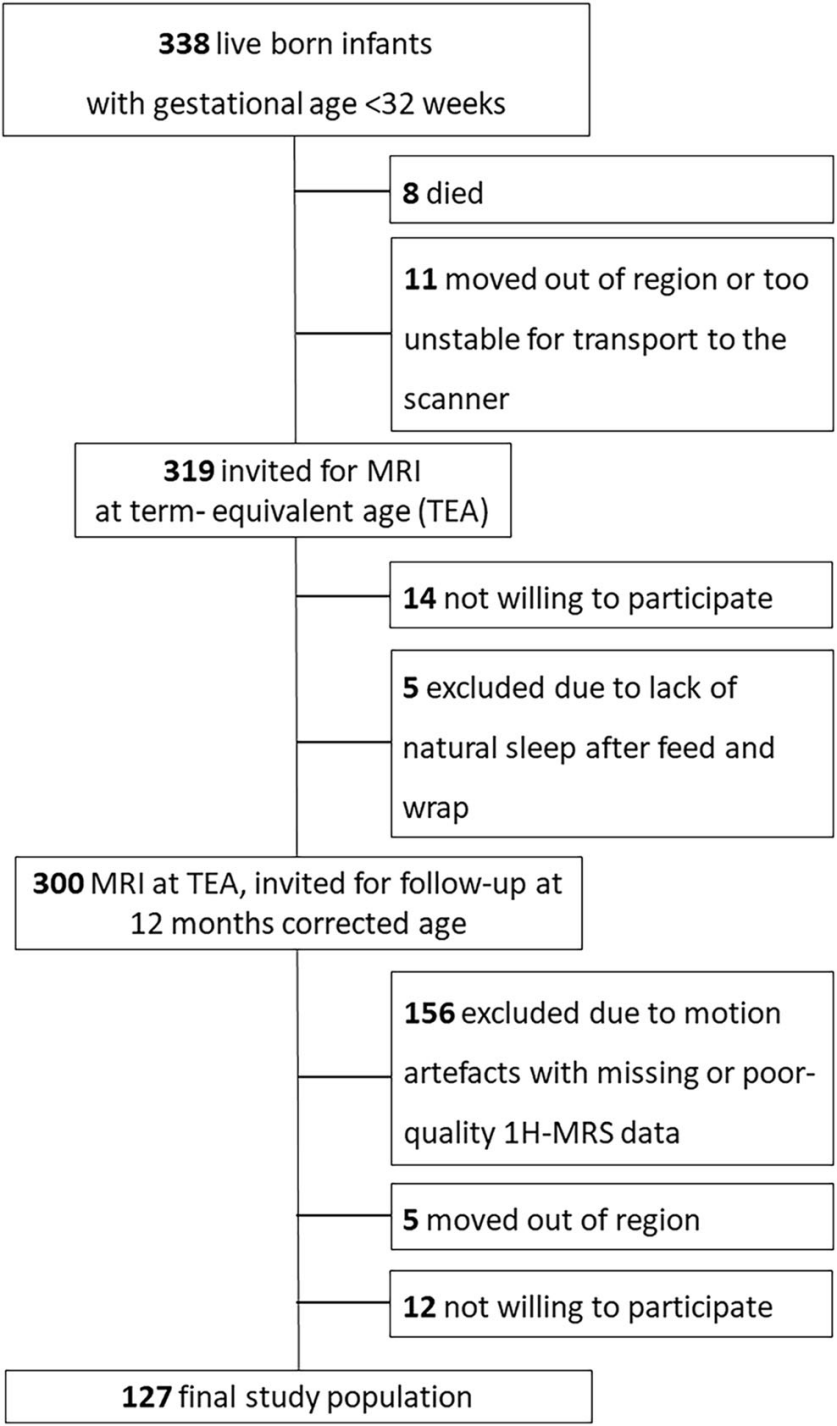
Flowchart of total study population with excluded and included infants

During the study period, 338 VPIs were born. Of those, 300 received an MRI scan at TEA during the study period and were invited for the clinical testing at 12 months corrected age. Because of the motion artefacts and consequently missing or poor-quality spectroscopy, 156 VPIs were excluded. After five neonates moved out of the region and the parents of 12 neonates were not willing to participate in the study, ^1^H-MRS and DTI data sets of 127 VPIs were included in the final data analysis (Fig. 1).

Relevant corrected ages of all 127 VPIs included in our study, with mean, median, standard deviation (SD) and range given in weeks were as follows: gestational age: 29.62, 29.86, 1.8 and 7.15; age at the time of MRI: 40.44, 40.43, 1.47 and 15.56; and age at the time of clinical testing for cognitive and motor development: 11.95, 12, 0.84 and 6.

At 12 months corrected age, the NDO of VPIs was assessed using the motor and cognitive scales of Bayley Scales of Infant and Toddler Development-Third Edition (Bayley-III) [19]. As previous studies showed that US norms for Bayley-III tend to underestimate developmental delay, we applied German norms in our study [23]. All VPIs with a score of less than 85 (> 1 SD below the mean) in either motor or cognitive scale were categorised as having a neurodevelopmental delay, according to standardised scores.

Further neonatal data are summarised in Table 1.

**Table 1:** Clinical characteristics of all included 127 preterm neonates

Median gestational age (weeks)	29.6 ± 1.8
Gender, female	58 (47.7%)
Small for gestational age	9 (7.1%)
Necrotizing enterocolitis	7 (5.7%)
Patent ductus arteriosus	28 (22%)
Surfactant	95 (74.8%)
Bronchopulmonary dysplasia (BPD)	24 (18.9%)
Postnatal steroid treatment for BDP	15 (11.8%)
Catecholamine treatment	7 (5.5%)
Sepsis, early onset	9 (7.1%)
Sepsis, late onset	13 (10.3%)

### MRI details

All MRI images were acquired using a 3-T Siemens Magnetom Verio scanner (Siemens) and “feed and wrap” technique without sedation [24]. Oxygen saturation of VPIs was monitored by a responsible neonatologist using a pulse oximeter. Special earmuffs were used for hearing protection. A 16-channel paediatric head coil was used for image acquisition.

A multivoxel, point resolved (PRESS) 2D chemical shift imaging (CSI) ^1^H-MRS was acquired at the level of supraventricular white matter with the following parameters: TE 135 ms, TR 1700 ms, FOV 160 × 160 mm, voxel size 10 × 10 × 15 mm and acquisition time 6.53 min.

The DTI images were acquired in the axial plane, covering the whole brain (matrix 160 × 160; *b* = 1000 s/mm^2^; DTI sequence with 20 directions repeated twice; TE 101 ms, TR 6600 ms, acquisition time 2:40 min).

The details on the remaining sequences from our protocol can be found in the supplementary material.

### Quantitative MRS and DTI analysis

The single metabolites’ spectra were evaluated in frontal white matter (left: FWML, right FWMR), central white matter (left: CWML, right: CWMR) and parietal white matter (left: PWML, right: PWMR) on both sides by using a dedicated software, jMRUI. The concertation of the following metabolites was quantified as the area under the curve of the corresponding peak: N-acetyl aspartate (NAA), choline (Cho), creatine (Cr) and myo-inositol (mI). After the spectra quantification, the following metabolite ratios were built: NAA/Cho, NAA/Cr, Cho/Cr, mI/Cr and NAA/mI.

**Fig. 2:**
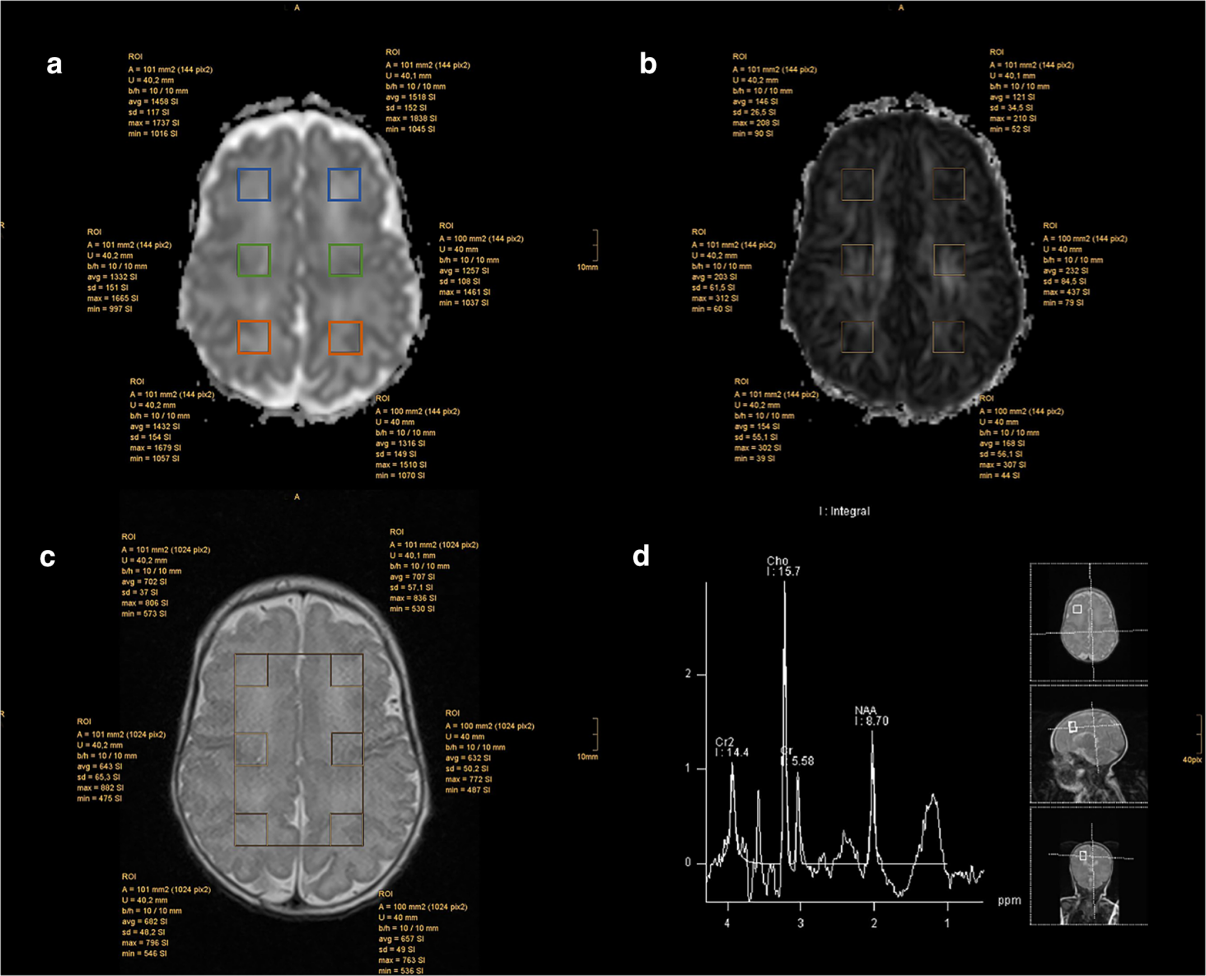
Regions of interest (ROIs) in all regions evaluated, positioned in MD (**a**), FA (**b**) and MRS localiser (**c**). Corresponding spectrum in frontal white matter on the right side (**d**). Frontal white matter right (FWMR) and left (FWML) in blue, central white matter right (CWMR) and left (CWML) in green and parietal white matter right (PWMR) and left (PWML) in orange

To analyse the DTI, the regions of interest (ROIs) were manually drawn in apparent diffusion coefficient maps and FA in the same voxels in which metabolites were measured, using an automatic correlation of the slices in the dedicated picture archiving and communication system Impax EE (Agfa Healthcare) (Fig. 2). The mean numbers and SD values for MD and FA were considered for further evaluation. FA is a unitless measure; MD has a unit of 10^−3^ mm^2^/s.

All measurements were performed by two independent rates (T.J. and V.W.) blinded for the results of the Bayley-III test.

### Evaluation of conventional MRI sequences

Conventional sequences were evaluated in the clinical routine by one neuroradiologist (T.J.) with extensive experience in neonatal imaging. The images were evaluated again for study purposes according to the Kidokoro Classification of brain injury [25], by two or three raters, blinded to the previous reports and NDO of the VPIs (T.J. and V.N./M.H.).

### Statistical analysis

Descriptive statistical analysis was performed with SPSS software version 26. Data distribution was evaluated by the Kolmogorov-Smirnov test. Group comparisons were performed by the Mann-Whitney *U* test. The interclass correlation coefficient (ICC) for interrater reliability was calculated by using a two-way mixed model. The significance level was set at 0.05.

### Feed-forward neural networks

**Fig. 3:**
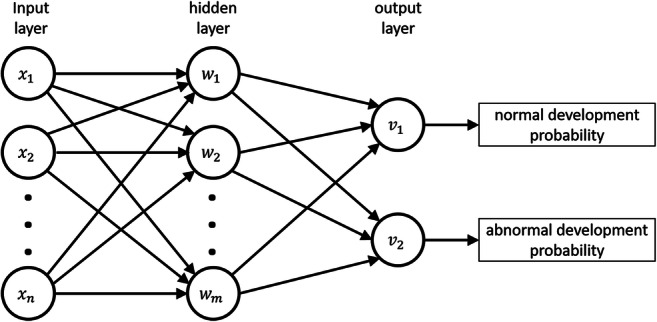
The network diagram represents the NNs employed in this study. We used single-hidden-layer feed-forward neural nets, as they are considered well suited for classification tasks [26]. NNs of this style consist of three layers: the input layer, the hidden layer and the output layer, where each unit of the input and hidden layer connects to each unit of the subsequent layer. By these connections, every unit of the hidden and output layer is a linear combination of all units of the preceding layer, followed by a nonlinear transfer function. The input units *x*_*i*_ correspond to the variables used for the class prediction, in this study metabolite ratios or DTI characteristics. The hidden units can be thought of as new derived variables that are not directly observable in the data. The output units represent the probabilities for the input characteristics to belong to a certain class

For the NDO prediction, we used single-hidden-layer feed-forward neural networks (fNNs) that are known to be highly flexible in their applications, allowing an approximation of nonlinear relationships between characteristics of an observation and its class (Fig. 3). Thus, the fNN functions have typically high predictive accuracy [16].

### Outcome prediction

First, a selection algorithm was constructed for receiving a subset of characteristics that were prevalent for the VPIs with developmental delay. This algorithm was constructed based on the level of the metabolite ratios and MD and FA values measured in delayed preterm neonates. Based on their distribution on an *x*-axis, localisation degrees (LD) of the measured values were determined. An LD ranges from 0.125 to 1, with low values corresponding to strong localisation and high values to weak localisation. Thus, a low LD value was an indicator of the characteristic selection. For example, if a metabolite ratio measured in cognitively delayed preterm neonates has an LD value of 0.125, then this means that all cognitively delayed preterm neonates have the values of this metabolite ratio very close to each other, namely, located within one octile of the range of the considered metabolite ratio. This LD-based characteristic selection can be considered as a more detailed version of the area-under-the-curve (AUC) characteristic selection.

Next, due to the existing imbalance between the high number of normal developed preterm neonates and the low number of developmentally delayed neonates, the actual prediction was performed as a two-step approach. Based on the above-described selection algorithm, in the first step, we built “developmental delay common relaxed zones” (DDCRZ), which contain characteristics’ values of the preterm neonates close to the corresponding values of the delayed neonates, selected on the basis of LD, and we analysed if characteristics of a VPI belonged to the DDCRZ. If characteristics of a VPI were in the DDCRZ, then they were further examined in the second step; otherwise, the VPI was categorised as normally developed. Further details are given in the supplementary material.

**Fig. 4:**
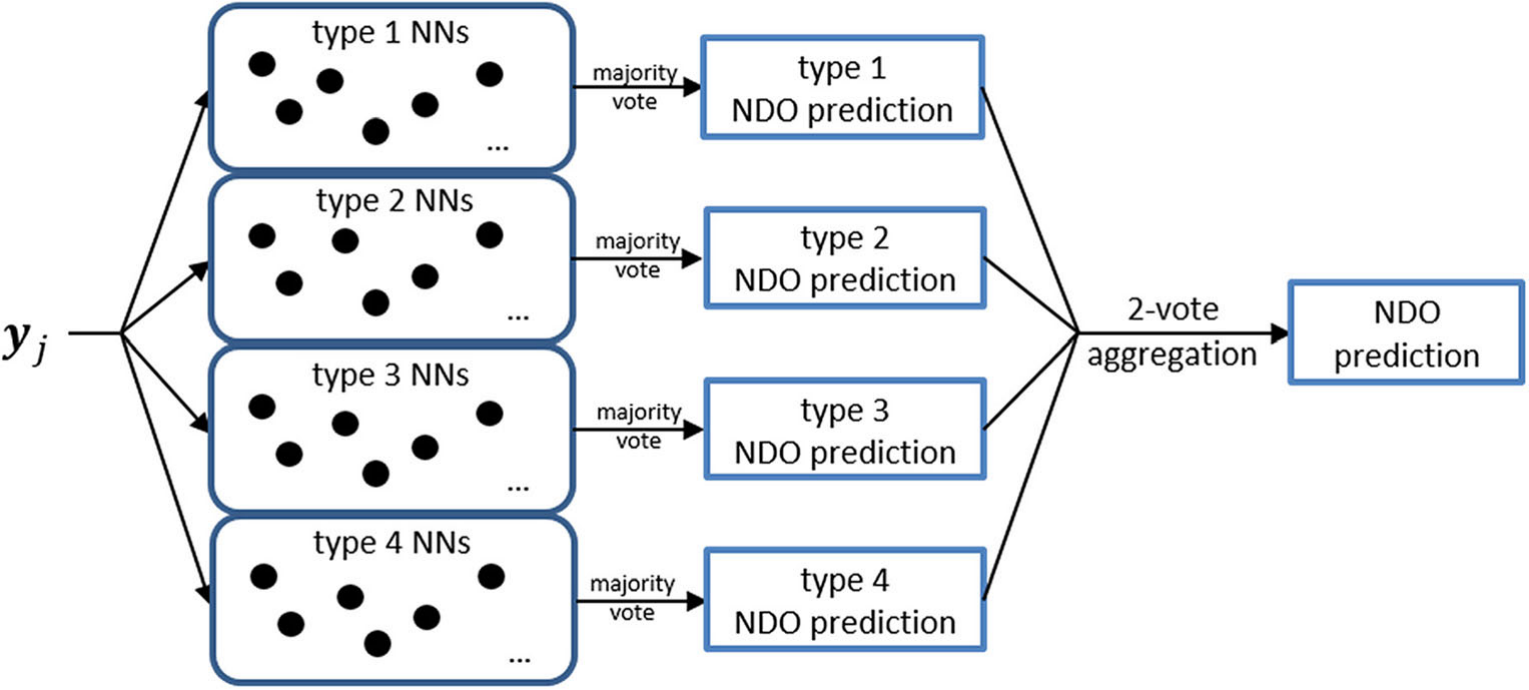
The illustration of the second step of the proposed predictor

In the second step, we employed a predictor consisting of several fNNs trained using a cross-validation technique. For this purpose, the data had been split fourfold. For each choice of a fold, fNNs have been trained on the data of the other folds with the chosen fold serving as validation data. We grouped networks of the same fold as of one ‘type’ and built 100 fNNs of each type to improve their ability of generalisation. The first prediction was performed by a majority vote within each fNN type. The final NDO prediction was formed by a two-vote aggregation of these answers (Fig. 4). Finally, sensitivity, specificity and positive and negative predictive values (PPV, NPV) were calculated.

An additional subanalysis was performed after excluding all VPIs with severe cerebral injury (grades 3 and 4).

In the supplementary file, we mathematically described further details of the above-mentioned methodical steps regarding fNNs.

## Results

### Patient characteristics

The main clinical characteristics of the VPIs are summarised in Table 1.

The NDOs dependent on the presence of brain injury are summarised in Table 2. A significant difference in motor outcome was found between the groups with and without periventricular leukoencephalopathy (*p* = 0.02).

**Table 2:** Brain injury and Bayley-III outcome of preterm neonates at 12 months corrected age

	Motor delay				Cognitive delay			
	Yes	No	Total	*p*	Yes	No	Total	*p*
PL								
Yes	6	15	21	0.02*	3	17	20	0.08
No	9	97	106		4	101	105	
Total	15	112	127		7	118	125	
IVH								
Yes	4	15	19	0.21	1	18	19	0.94
No	11	97	109		6	100	106	
Total	15	112	127		7	118	125	
CBH								
Yes	2	11	13	0.68	0	13	13	0.21
No	13	101	114		7	105	112	
Total	15	122	127		7	118	125	

*PVL* periventricular leukoencephalopathy, *IVH* intraventricular haemorrhage, *CBH* cerebellar haemorrhage

*Significance level 0.05

The ICCs for the quantification of MRS with JMRUI were 0.99–1, for MD 0.81–0.89 and FA 0.8–0.87. A mean value of both measurements for MD and FA was used for further calculations.

The ICCs for the evaluation of conventional MRI were 0.93 for PVL, 0.97 for IVH and 0.99 for CBH.

### MRS and DTI measurements

Mean values and SDs of the metabolite ratios, and MD and FA for delayed and non-delayed groups are listed in Tables 3, 4 and 5, respectively. The MRS values are comparable with those of previously published studies, which employed 135 ms TE [27–29].

**Table 3:** Mean (SD) metabolite ratios in frontal (FWM), central (CWM) and parietal (PWM) supraventricular white matter on both sides (right R and left L) according to the motor outcome at 12 months corrected age

	Motor Bayley-III < 85					Motor Bayley-III > 85				
	NAA/Cho	NAA/Cr	Cho/Cr	NAA/ml	ml/Cr	NAA/Cho	NAA/Cr	Cho/Cr	NAA/ml	ml/Cr
FWMR	0.6 (0.12)	1.23 (0.31)	2.05 (0.39)	2.47 (0.68)	0.49 (0.18)	0.62 (0.1)	1.33 (0.36)	2.16 (0.45)	2.44 (0.76)	0.53 (0.19)
FWML	0.66 (0.17)	1.29 (0.55)	1.93 (0.51)	2.82 (0.99)	0.46 (0.13)	0.61 (0.14)	1.28 (0.4)	2.13 (0.49)	2.37 (0.95)	0.55 (0.19)
CWMR	0.76 (0.15)	1.34 (0.25)	1.76 (0.21)	3.28 (0.89)	0.42 (0.08)	0.61 (0.15)	1.28 (0.29)	2.13 (0.25)	2.37 (1.42)	0.55 (0.14)
CWML	0.77 (0.09)	1.39 (0.19)	1.81 (0.14)	3.30 (0.53)	0.43 (0.05)	0.78 (0.01)	1.39 (0.21)	1.79 (0.25)	3.65 (1.32)	0.37 (0.13)
PWMR	0.95 (0.22)	1.64 (0.33)	1.61 (0.39)	3.79 (1.42)	0.37 (0.12)	0.92 (0.22)	1.61 (0.43)	1.68 (0.44)	3.75 (1.64)	0.37 (0.15)
PMWL	0.87 (0.1)	1.55 (0.32)	1.74 (0.28)	3.89 (0.71)	0.41 (0.07)	0.92 (0.16)	1.59 (0.32)	1.74 (0.28)	3.92 (1.53)	0.35 (0.15)

**Table 4:** Mean (SD) metabolite ratios in frontal (FWM), central (CWM) and parietal (PWM) supraventricular white matter on both sides (right R and left L) according to the cognitive outcome at 12 months corrected age

	Cognition Bayley-III < 85					Cognition Bayley-III > 85				
	NAA/Cho	NAA/Cr	Cho/Cr	NAA/ml	mI/Cr	NAA/Cho	NAA/Cr	Cho/Cr	NAA/ml	mI/Cr
FWMR	0.64 (0.14)	1.47 (0.31)	2.32 (0.48)	2.7 (0.77)	0.57 (0.16)	0.61 (0.1)	1.31 (0.36)	2.14 (0.44)	2.41 (0.74)	0.52 (0.19)
FWML	0.61 (0.19)	1.18 (0.56)	1.88 (0.62)	2.58 (1.2)	0.48 (0.19)	0.61 (0.15)	1.29 (0.41)	2.13 (0.49)	2.41 (0.96)	0.54 (0.19)
CWMR	0.82 (0.15)	1.42 (0.28)	1.75 (0.25)	3.82 (1.4)	0.36 (0.14)	0.72 (0.14)	1.28 (0.22)	1.78 (0.13)	3.21 (0.94)	0.41 (0.08)
CWML	0.80 (0.14)	1.42 (0.29)	1.79 (0.13)	3.82 (1.4)	0.36 (0.14)	0.72 (0.14)	1.28 (0.22)	1.78 (0.13)	3.21 (0.94)	0.41 (0.08)
PWMR	0.89 (0.14)	1.56 (0.13)	1.76 (0.19)	4.13 &1.07)	0.39 (0.07)	0.92 (0.23)	1.61 (0.43)	1.68 (0.44)	3.71 (1.65)	0.37 (0.15)
PWML	0.88 (0.10)	1.55 (0.21)	1.76 (0.19)	3.96 (0.72)	0.39 (0.06)	0.92 (0.15)	1.59 (0.32)	1.74 (0.28)	3.92 (1.51)	0.36 (0.15)

**Table 5:** Mean (SD) MD and FA values in frontal (FWM), central (CWM) and parietal (PWM) supraventricular white matter on both sides (right R and left L) according to the motor and cognitive outcome at 12 months corrected age. FA is unitless; MD unit is 10^−3^ mm^2^/s

	Motor Bayley-III < 85		Motor Bayley-III > 85		Cognition Bayley-III < 85		Cognition Bayley-III > 85	
	MD	FA	MD	FA	MD	FA	MD	FA
FWMR	1.33 (0.11)	1.16 (0.03)	1.42 (0.15)	0.16 (0.02)	1.36 (0.12)	0.18 (0.03)	1.14 (0.15)	0.16 (0.02)
FWML	1.31 (0.11)	0.16 (0.03)	1.04 (0.15)	0.15 (0.02)	1.35 (0.12)	0.17 (0.04)	1.39 (0.15)	0.15 (0.02)
CWMR	1.26 (0.16)	0.22 (0.04)	1.32 (0.16)	0.23 (0.04)	1.31 (0.15)	0.23 (0.05)	1.32 (0.15)	0.23 (0.04)
CWML	1.28 (0.18)	0.23 (0.05)	1.32 (0.16)	0.23 (0.04)	1.31 (0.2)	0.23 (0.07)	1.31 (0.16)	0.23 (0.04)
PWMR	1.31 (0.12)	0.17 (0.03)	1.4 (0.15)	0.17 (0.03)	1.39 (0.22)	0.18 (0.05)	1.39 (0.14)	0.17 (0.03)
PWML	1.34 (0.18)	0.17 (0.04)	1.36 (0.13)	0.17 (0.03)	1.37 (0.23)	0.18 (0.04)	1.36 (0.13)	0.17 (0.03)

### Motor development prediction

Out of 300 VPIs who received an MRI scan at TEA, 278 could be evaluated at 12 months corrected age for their motor skills. Out of 127 included VPIs, 24 were excluded in a second step due to some missing metabolite peaks. In total, 103 complete MRS datasets of VPIs for the prediction of motor delay were available. Of those, 13 (12.6%) infants were categorised as motorically delayed at 12 months corrected age. There was no difference in the rate of neurodevelopmental delay (12.6 vs. 17.1%, *p* = 0.391) or the mean scores of motor scales (100 ± 16.3 vs. 97 ± 17.5, *p* = 0.226) between included and excluded infants.

In view of their low LD values, the following characteristics were classified as significant for the motor delay with the above-described approach: NAA/Cho (CWMR), NAA/mI (CWMR), mI/Cr (CWMR), mI/Cr (CWML), NAA/Cho (PWMR), Cho/Cr (PWMR) and NAA/Cr (PWML). Due to their stronger localisation properties, the later five characteristics were used in the refined second step of our algorithm as inputs for the fNNs for predicting the motor outcome of VPIs in the DDCRZ.

The proposed fNN-based predictor could achieve the prediction of motor outcome with a sensitivity of 100%, a specificity of 90.9%, a PPV of 90.9% and an NPV of 100%. Since three motorically delayed VPIs were not included in the DDCRZ (i.e. these VPIs were classified as normally developed in the first step of our predictor), the performance of the proposed predictor on the whole data set was modified as follows: 76.9% sensitivity, 98.9% specificity, 90.9% PPV and 96.7% NPV.

In the subanalysis, after excluding all VPIs with high-grade cerebral injury, a sensitivity of 100%, a specificity of 87.5%, a PPV of 88.9% and an NPV of 100% were achieved. After the correction due to the loss of three VPIs in the DDCRZ, the results were corrected as follows: 72.7% sensitivity, 98.9% specificity, 88.9% PPV and 96.6% NPV.

### Cognitive development prediction

Out of 300 VPIs, 276 VPIs could be evaluated regarding their cognitive development at 12 months corrected age.

Out of 127 included VPIs, 12 were excluded in the second step due to some missing metabolite peaks. In total, 115 complete datasets of VPIs for the prediction of cognitive outcome were available. Among them, seven (6.1%) VPIs were identified as having a cognitive delay at the corrected age of 12 months. The proportion of cognitively delayed VPIs was similar to the excluded group, nine of 161, 5.6% (*p* = 1.000). Again, there was no difference in the mean score for the cognitive scale of Bayley-III between included and excluded infants (104 ± 15.1 vs. 102 ± 16.8, *p* = 0.290).

The following metabolite ratios and diffusion characteristics showed low LD values and were therefore used for constructing the DDCRZ for cognitive delay estimation: NAA/Cho (FWMR), Cho/Cr (FWMR), Cho/Cr (FWML), mI/Cr (FWML), NAA/Cr (FWMR), NAA/mI (FWMR) and FA (FWMR). Analogously to the motor case, due to their stronger localisation properties, the latter four characteristics were used as the inputs for the fNNs at the refined second step of our algorithm.

With the employed fNNs, we achieved a sensitivity of 85.7%, a specificity of 100%, a PPV of 100% and an NPV 99.1% (93.3% in DDCRZ). After excluding the VPIs with severe brain injuries visualised on conventional MRI (two of seven), all statistical parameters were by 100%.

## Discussion

In the present study, we were able to predict the NDO in VPIs at 12 months corrected age by employing fNNs and using biomarkers of ^1^H-MRS and DTI. Using the metabolite ratios of mI/Cr, NAA/Cr and NAA/mI and FA values quantified in frontal white matter, we predicted cognitive delay with a sensitivity of 85.6% and a specificity of 100%. At the same time, we achieved the prediction of motor delay with a sensitivity of 76.9% and a specificity of 98.9% using NAA/Cho, Cho/Cr, NAA/Cr and mI/Cr measured in central and parietal white matter. As far as we are aware, this is the first study that combines ^1^H-MRS metabolite ratios and DTI values for constructing early outcome prognostic biomarkers by employing fNNs. Thus, we obtained a higher predictive accuracy than qualitative conventional MRI or other statistical methods published so far [28, 30–32].

DTI is used for assessment of microstructural white matter development including myelination, fibre bundle integrity and connectivity of axons by quantifying MD and FA [33]. Highly organised tissue, such as cell membranes and myelin, directionally restricts the diffusivity of the molecular water measured by DTI [34]. The degree of directional heterogeneity measured by FA reflects microstructural fibre tract development, including the number, size and myelination of axons [34]. On the other hand, a lower MD mirrors higher spatial anisotropy of water diffusion and increased fibre tract development [34]. With advanced brain development, FA, therefore, increases and MD decreases, reflecting greater fibre organisation and preliminary myelination [35, 36]. At the same time, progressive brain maturation and myelination result in changes of the white matter metabolism, primarily regarding NAA and Cho concentration [37–40]. NAA is synthesised in neuronal mitochondria and its concentration increases markedly in the neonatal period [14, 37, 41], supposedly due to the increased employment of acetyl groups for lipid synthesis and myelin storage [42]. Moreover, NAA contributes to the metabolism of brain fatty acids, osmoregulation and neuromodulation. At the same time, the concentration of choline decreases due to its gradual incorporation into the myelin-associated macromolecules, making it invisible for MRS [37, 43]. Consequently, metabolite ratios incorporating NAA and Cho represent effective biomarkers of myelination.

The central pathological feature of the white matter injury of prematurity is the predisposition for myelination failure due to arrested pre-oligodendrocyte maturation and their inability to generate oligodendrocytes [44]. Diffuse reactive astrogliosis and, to a lesser extent, axonopathy in the form of microscopic necrosis are the consequences, which cannot be precisely depicted in conventional MRI [44]. Hence, MRS and DTI are suitable for better characterisation of the severity of diffuse astrogliosis and microscopic necrosis in preterm survivors.

The density of susceptible immature oligodendrocytes varies significantly between brain regions and is shown to be much higher in the frontal and posterior periventricular white matter [45–47]. That makes these regions particularly vulnerable to the WMI of prematurity [48]. At the same time, due to the origin of the executive functions and higher-order cognition in the frontal lobe [49], this region is of particular interest in the cognitive development of preterm neonates [50, 51]. On the other hand, due to a relevantly increased risk for the development of cerebral palsy in preterm infants [52], the corticospinal tract is the most frequently researched white matter tract in this population [53]. Therefore, constituting a relevant portion of the corticospinal tract, the central white matter (centrum semiovale) represents another essential area of interest in preterm neonates. We were able to assess all these relevant areas at once by placing a multivoxel ^1^H-MRS at the level of the supraventricular white matter. Commensurate with previous findings, FA values and metabolite ratios assessed in frontal white matter were of significant relevance for the prediction of cognitive outcome in our cohort. Accordingly, in a recently published study, Guo et al demonstrated that the only region relevant to the prognosis of cognitive outcome was FWM [50]. On the other side, by employing our methods, the metabolic and microstructural composition of central and parietal/posterior white matter played an important role for motor outcome prediction, which is in line with the literature [28].

We found a significant impact of WMI detected on conventional sequences on the motor outcome, according to the previous conventional MRI studies in preterm infants [4, 6]. However, the impact of subtle brain injury has been increasingly emphasised in quantitative MRI studies [54–56] and the outcome of infants having those remains uncertain [57]. As mild WMI can resolve over time [50], conventional MRI at TEA may underestimate their burden. Also, even though the severity of premature related WMI has been shifted from extensive cystic to subtle diffuse and punctate WMI due to advances in neonatal intensive care [58], no corresponding improvements in NDO or prevalence reduction of non-cystic WMI have been observed [59]. Therefore, the disruption of myelination and white matter connectivity assessed by DTI and MRS quantification is a more reliable method for evaluation of the premature white matter [30]. Moreover, quantitative assessment of cerebral microstructure and metabolism in preterm neonates is more strongly associated with NDOs than simple qualitative evaluation of WMI [11, 12].

To the best of our knowledge, so far only three studies have combined ^1^H-MRS and DTI measured in VPIs at TEA with the aim of ruling out any association with their NDO [27, 28, 31]. However, only one study intended to determine prognostic biomarkers by this method [28]. Kendall et al examined 43 VPIs at TEA with ^1^H-MRS and DTI with the purpose of determining biomarkers of the motor outcome. Similar to our results, they succeeded to predict motor development using Cho/Cr and NAA/Cho ratios measured in parietal white matter. However, the evaluated DTI data was not considered for biomarker construction and no prediction of cognitive development was achieved.

Apart from combining ^1^H-MRS and DTI to increase the accuracy of the prognostic value of MRI, we applied a complex statistical method for ruling out the prognostic biomarkers. The first part of the proposed predictor has a structure of a classification tree [60], a commonly used diagnostic tool [61]. The algorithm for its construction, which uses the LDs, is similar to the CART algorithm for the construction of classification trees [62]. The involved fNNs are trained on several different validation and training subsets, to construct a predictor able to generalise new data as best as possible. Therefore, high accuracy on additional data is expected as well, rendering the proposed method as clinically applicable.

The strength of our study consists of its prospective character, a relatively large cohort and a sophisticated novel statistical method. However, some limitations should be named as well.

First, the VPIs’ outcomes in our study were assessed at 12 months corrected age, as in some studies [28], although most previous studies reported outcomes at 18–24 months. However, as the developmental categorisation at this later stage may still not necessarily reflect the outcome at an older age [63, 64], a follow-up study at 5 years corrected age would be more accurate, which we plan to do. Second, despite a relatively large sample size, the rate of motorically and cognitively delayed VPIs is lower than in other studies [28, 31]. This, at least to some extent, might reflect efficient intensive care interventions applied in our institution. Another reason could be the clinical test applied (Bayley-III), which was reported to possibly overestimate the outcome of preterm neonates in comparison with the BSID-II. However, we employed German norms, which were shown to overestimate the outcome to a lesser extent when compared to US norms [23]. Moreover, we tried to deal with this issue of imbalanced data by a suitable approach as recommended previously [65, 66]. Third, due to the methods applied, we faced a classification error in the first step of our predictor in the case of the motor outcome, missing such three of our 13 motorically delayed neonates. When we receive the data of further preterm neonates, we are planning to consider various possibilities for reducing this error in a further study. These include, for example, the building of the DDCRZ using the ideas of the nearestneighbours’ classifiers [67] and consideration of various covariates in our predictors. Apart from that, the prediction result could be extended to include a category of ‘risky development’. The VPIs with this development would be predicted to be at risk of developmental delay. This extension may allow an additional prediction improvement because the delayed VPIs that are predicted to be normal by the binary predictor (i.e. a predictor that distinguishes between normal and delayed VPIs) could be predicted to be at risk for developmental delay by the extended predictor. Fourth, our methodology does not suggest to use any DTI characteristics for the prediction of the motor outcome. However, for the cognitive outcome prediction, one DTI characteristic, namely FA measured in frontal white matter on the right side, plays an important role and has a low localisation degree, and it is therefore used by the fNNs in the second prediction step. Some additional DTI characteristics might, however, become important in our further methodology as discussed above. Fifth, even though we employed a multivoxel ^1^H-MRS with 135 ms TE, we quantified mI at 3.56 ppm. MI peak is the dominant peak in short TE ^1^H-MRS, and its height is reduced in intermediate TE MRS [37]. Consequently, in a neglected number of MRS spectra assessed, the mI peak was not quantifiable. In addition, due to the elimination of the mI multiplet, a contribution of Gly in this peak has to be considered in this context. Finally, even though we included 127 infants in our study, the dropout rate is quite high considering that 300 infants received an MRI. This results primarily from the combination of the sequence order in our MRI protocol and the absence of sedation. Namely, ^1^H-MRS was performed as the very last sequence in our protocol and, with the feed and wrap technique, not all neonates were able to make through the complete MRI examination.

In conclusion, fNNs might be a utile predictive tool for cognitive and motor outcome prediction of VPIs at 12 months corrected age when employing early biomarkers of cerebral ^1^H-MRS and DTI evaluated at TEA. Our findings may have implications for clinical practice in spotting those VPIs that would most benefit from early intervention services and neuroprotective care. The proposed approach could be applied as a complemental predictive tool, particularly in VPIs that show no or mild cerebral injury on conventional MRI, whose NDO, therefore, remains uncertain.

## Electronic supplementary material

ESM 1 (DOCX 612 kb)

## Abbreviations

^1^H-MRS: Proton magnetic resonance spectroscopy
Cho: Choline
Cr: Creatine
FA: Fractional anisotropy
fNNs: Feed-forward neural networks
MD: Mean diffusivity
mI: myo-Inositol
NAA: N-Acetyl-aspartate
NDO: Neurodevelopmental outcome
TEA: Term-equivalent age
VPIs: Very preterm infants
WMI: White matter injury
