# Supplementary material for: Feed-forward neural networks using cerebral MR spectroscopy and DTI might predict neurodevelopmental outcome in preterm neonates
Source: Eur Radiol. 2020 Jul 18;30(12):6441–51. doi: 10.1007/s00330-020-07053-8 (PMC7599175; doi:10.1007/s00330-020-07053-8)
Supplement: Supplementary file 1 — (DOCX 612 kb) [file 330_2020_7053_MOESM1_ESM.docx]

**Supplementary material**

**Conventional MRI sequences from our protocol**

Apart from DTI and ^1^H-MRS, the following sequences were employed as a part of our standardized institutional protocol:

- T2 TSE (TE 99 ms, TR 4590 ms, flip angle 150, FOV 150 x 108 mm, matrix 256 x 205, slice thickness 3 mm, distance factor 0%) in the axial plane
- 3D T1 MP-RAGE (TE 4.54 ms, TR 1770 ms, TI 1000 ms, flip angle 9 degrees, FOV 200 x 150 mm, matrix 144 x 192, slice thickness: 1.0 mm, gap 0.5 mm) in the sagittal plane
- Susceptibility-weighted images (SWI) (TE 20 ms, TR 27 ms, FOV 200 x 150 mm, matrix 182 x 256, slice thickness: 2.0 mm, gap: 0.4 mm) in the axial plane.

**Statistical analysis with fNNs**

We consider the problem of predicting NDO using VPIs MRS and DWI characteristics$\boldsymbol{x}=(x_{1}, x_{2},\ldots,x_{n})$ in the framework of the statistical classification [1]. In this framework, we construct a predictor that using the VPIs characteristics $\boldsymbol{x}$, or a suitably selected part of them, gives a prediction of the VPIs NDO. Before constructing this predictor, it is reasonable to perform characteristic selection [2].

We consider characteristic selection that is based on the characteristic ability to localize the VPIs with developmental delay. As a localization measure, we consider the localization degree (LD) of a characteristic $x_{i}$ defined as follows. Let $\bar{N}$ be the number of the developmentally delayed VPIs, and $N_{i}$ be the number of octiles of the $x_{i}$-distribution that contains bigger than $\bar{N}/8$ $x_{i}$-values that belong to the underdeveloped VPIs. Then, the LD of $x_{i}$ is defined as ${LD}_{i}=N_{i}/8$.

The LD has values in the interval [0.125, 1] with low values corresponding to a strong localization, and with high values ― to weak localization. Thus, a low LD value can be seen as an indicator for the characteristic selection. We demonstrate the calculation and the meaning of the LD in Figure 1.

Assume that some characteristics have been selected, and let us denote them as $\boldsymbol{y}=\left( y_{1},y_{2},\ldots,y_{m} \right)$. For predicting the developmental delay, we propose a 2-step predictor.

**The first step of predictor**

For the first step, we build a “developmental delay common relaxed zone” (DDCRZ). This zone is used for selecting characteristics vectors $\boldsymbol{y}_{j}$ that are suspected to belong to a VPI with a developmental delay. This selection is done in the following manner:

- If the characteristics vector $\boldsymbol{y}_{j}$ for the j-th VPI belongs to the DDCRZ, then this VPI is suspected to be delayed, and its characteristics vector $\boldsymbol{y}_{j}$ is analyzed further in the second step.
- Otherwise, the j-th VPI is predicted to have normal development.

This first step of our predictor is illustrated in Figure 2.

The DDCRZ is constructed in a way that it contains characteristics vectors $\boldsymbol{y}$ that are close to the characteristic’s vectors $\boldsymbol{y}_{j_{l}}$ of the delayed VPIs. This zone is needed due to the imbalanced class distribution in our data. Namely, there are many VPIs with normal NDO, and only a few VPIs have an impaired NDO. For the imbalanced data, a contraction of the data set is needed, such that the class distribution in the contracted data set becomes considerably more balanced [3, 4]. Our DDCRZ realizes this contraction as follows.

First, we form the so-called developmental delay zone ${DDZ}_{i}$ for each selected characteristic $y_{i}$. The ${DDZ}_{i}$ consists of octiles of the $y_{i}$-distribution that contain bigger than $\bar{N}/8$ $y_{i}$-values that belong to the VPIs with the developmental delay (see Figure 1).

Then, we add to the ${DDZ}_{i}$ the hexadeciles of the $y_{i}$-distribution that are located near the octiles in the ${DDZ}_{i}$. In this way, we obtain the developmental delay relaxed zone ${DDRZ}_{i}$ (Figure 3).

Using the ${DDRZ}_{i}$, the DDCRZ is finally built as follows:

$$DDCRZ=\left\{ \begin{aligned} \boldsymbol{y}=\left( y_{1},y_{2},\ldots,y_{m} \right) such that y_{1}\in{DDRZ}_{1}, y_{2}\in{DDRZ}_{2},\ldots,y_{m}\in{DDRZ}_{m} \end{aligned} \right\}.$$

In addition to the LD, the structure of the DDCRZ has also an influence on the characteristic selection. First of all, it must be noted that if $\bar{N}\geq8$, then some delayed VPIs may be missing in the DDCRZ. Our cognitive data set contains $\bar{N}=7<8$ underdeveloped VPIs. In this case, there is no loss of the delayed VPIs in the DDCRZ. Let us begin with the consideration of this case.

**Cognitive case**

The characteristics in this case have the following LD values in the increasing order: 0.375, 0.5, 0.625, 0.75. The LD=0.375 belongs only to mI/Cr (FWML). Two characteristics, NAA/mI (FWMR) and FA (FWMR), have the LD=0.5. These characteristics are taken to the selected characteristics for the cognitive DDCRZ.

Seven characteristics have the next LD value of 0.625. For selecting characteristics among these characteristics, we use the relaxed LD (RLD). The ${RLD}_{i}$ of a characteristic $y_{i}$ is defined as follows. Let ${RN}_{i}$ be the number of the hexadeciles in the ${DDRZ}_{i}$, then ${RLD}_{i}={RN}_{i}/16$. In Figure 1, for illustration of the RLD calculation, we present the RLD values for the considered characteristics there.

For the characteristics with LD=0.625, the RLD values lie in the interval [0.8125, 1]. Four of these characteristics (namely, NAA/Cho (FWMR), Cho/Cr (FWMR), Cho/Cr (FWML), NAA/Cr (FWMR)) have the smallest RLD value 0.8125, and therefore, they are further taken to the selected characteristics for the cognitive DDCRZ.

With the selected characteristics, the cognitive DDCRZ contains in total 21 VPIs including seven cognitively delayed VPIs. Increasing the cut-off values for the LD and RLD does not lead to the further contraction of the DDCRZ, whereas their decrease leads to the increase of the number of VPIs in the DDCRZ, which then does not allow accurate identification of the delayed VPIs with fNNs in the second prediction step. Thus, in this way chosen cut-off values are optimal.

**Motor case**

In our motor data set, there are $\bar{N}=13\geq8$ delayed VPIs. In this case, some delayed VPIs may be missing in some ${DDRZ}_{i}$. Then these delayed VPIs are also missing in the DDCRZ that leads to a prediction error of the first step of our predictor.

In order to reduce this error, the loss of delayed VPIs needs to be incorporated into the characteristic selection. For this purpose, we consider the quantity $\bar{NC}_{i}$ that is the number of the delayed VPIs whose $y_{i}$-values are not located in the corresponding ${DDRZ}_{i}$. For an illustration of this quantity, in Figure 1, we present the distribution of the characteristic NAA/Cho (PWMR) that has $\bar{NC}=1$.

Among the characteristics in the motor case, there are four characteristics (namely, NAA/Cho (CWMR), mI/Cr (CWMR), mI/Cr (CWML), NAA/mI (CWMR)) for which $\bar{NC}_{i}=0$. Their LD values are also low (correspondingly, 0.625, 0.5, 0.5, 0.625). Thus, these characteristics are taken to the selected characteristics for the motor DDCRZ.

Further, there are 6 characteristics with $\bar{NC}_{i}=1$. Among them, there is NAA/Cho (PWMR) with a low LD=0.375, and there are Cho/Cr (PWMR) and NAA/Cr (PWML) with LD=0.5 and RLD=0.75. Other characteristics with $\bar{NC}_{i}=1$ and LD=0.5 have higher RLD values. Consequently, the mentioned characteristics are further taken to the selected characteristics for the motor DDCRZ.

With the selected characteristics, the motor DDCRZ has a balanced class distribution: it contains in total 21 VPIs including 10 motorically delayed VPIs. Threemotorically delayed VPIs are missing in the constructed DDCRZ, and therefore, these VPIs are misclassified as normally developed VPIs in the first step of our predictor. Thus, the choice of the cut-off values for the LD and RLD is controlled by the need to have a balanced class distribution in the DDCRZ, and also to have a low number of the missing developmentally delayed VPIs in the DDCRZ.

**The second step of predictor**

In the second step, for predicting the NDO of the VPI with the characteristic vector $\boldsymbol{y}\in\mathrm{DDCRZ}$, we use the feedforward neural networks (fNNs). For this purpose, for avoiding the overparameterization of the fNN model, from the characteristics $\boldsymbol{y}$, we first select a subset of characteristics $\bar{\boldsymbol{y}}=\left( \bar{y}_{1},\bar{y}_{2},\ldots,\bar{y}_{\bar{m}} \right)$. For the characteristic selection, we use the localization properties of the characteristics, and we select the characteristics with stronger localization.

In the cognitive case, we select the characteristics with low LD≤0.5: mI/Cr (FWML), NAA/mI (FWMR), FA (FWMR). The DDZ for the characteristics in the cognitive $\boldsymbol{y}$ with LD=0.625 has two connected octile components. For the characteristic NAA/Cr (FWMR), one of these components consists of only one octile, while these components for other characteristics in the cognitive $\boldsymbol{y}$ with LD=0.625 have always more than one octile. Therefore, NAA/Cr (FWMR) was also taken to the characteristics in the cognitive $\bar{\boldsymbol{y}}$.

In the case of the motor outcome, we first select the characteristics with $\bar{NC}_{i}=1$: NAA/Cho (PWMR), Cho/Cr (PWMR), NAA/Cr (PWML). Then we add the characteristics with $\bar{NC}_{i}=0$ and ${LD}_{i}=0.5$: mI/Cr (CWMR) and mI/Cr (CWML).

After the subset $\bar{\boldsymbol{y}}$ of characteristics is selected, the vector $\bar{\boldsymbol{y}}$ is analyzed by the fNNs. We use the fNNs with one hidden layer that are available in the MATLAB program (version R2018a, The MathWorks, Natick, Massachusetts, USA). The number of units in the hidden layer was equal to the number of characteristics in $\bar{\boldsymbol{y}}$, and the cross-entropy loss function was used.

We employed the stratified four-fold cross-validation technique for the construction of the fNNs. In this technique, four different types of fNNs are constructed. Due to the stochastic nature of the employed optimization algorithm, the construction of an fNN is usually nonunique. Therefore, to reduce the dependence on a particular realization of the used random optimization procedure, we run it 100 times for each of the four considered training subsets in the cross-validation technique. In this way, we obtain four types of fNNs consisting of 100 fNNs each.

The second step of our predictor operates as follows. The characteristic vector $\bar{\boldsymbol{y}}$ is given to the constructed fNNs for the NDO prediction. The answers of the fNNs of the same type are then aggregated using the majority vote. The final NDO prediction is formed by the two-vote aggregation of these answers. We illustrate this step of our predictor in Figure 4.

**Figures**


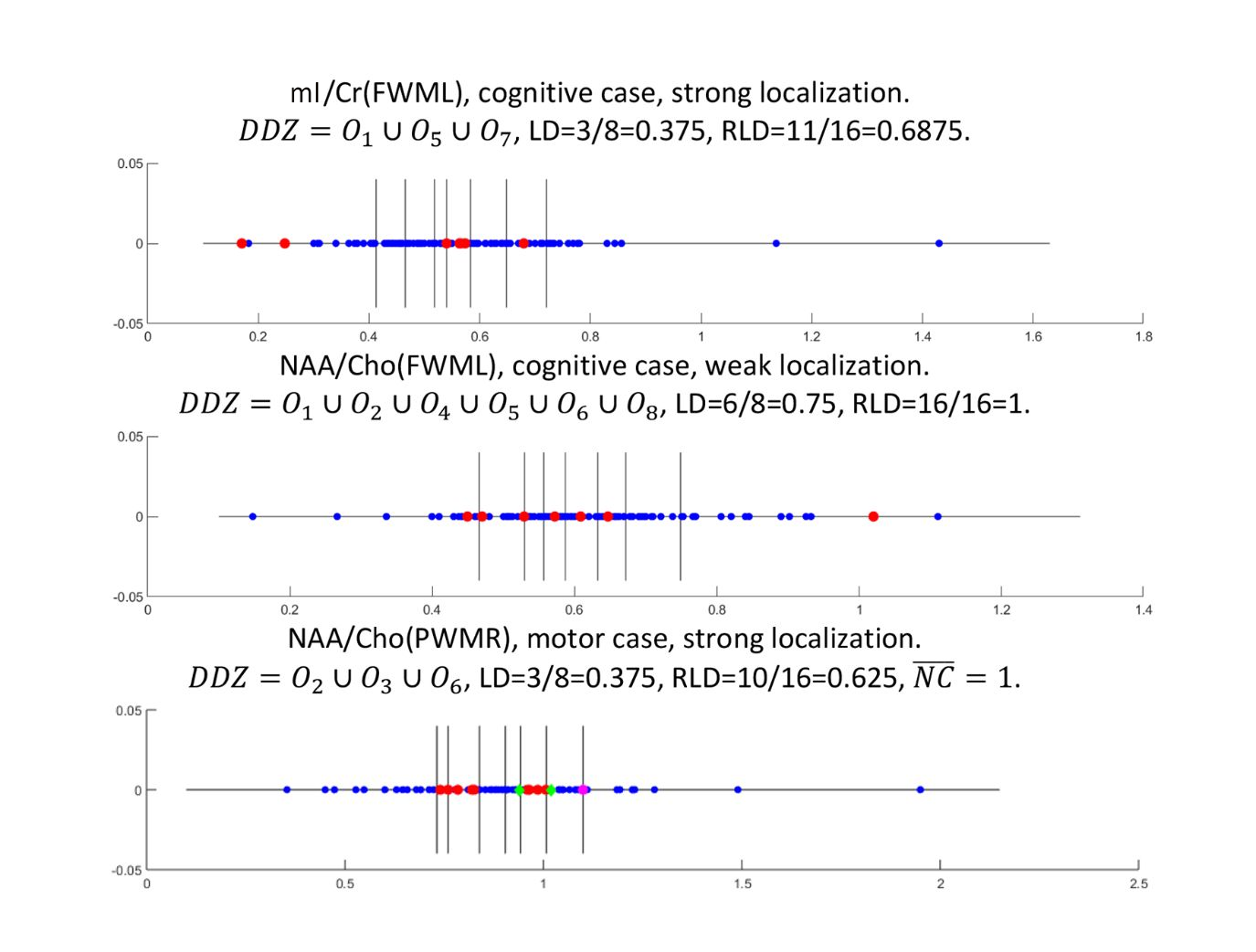


Figure 1. Examples of the determination of developmental delay zone (DDZ), localization degree (LD) and relaxed localization degree (RLD) for the distributions of three characteristics. The vertical lines show the octile points. The blue points show the characteristic values that belong to VPIs with the normal NDO, while the red points show the characteristic values that belong to VPIs with the delayed NDO. In the case of NAA/Cho measured in parietal white matter on the right side (PWMR), there are also two green diamond points and one magenta point that show the characteristic values that belong to VPIs with the delayed NDO. The green points show the characteristic values that do not belong to the corresponding DDZ but are still located in the corresponding DDRZ, whereas the magenta point shows the characteristic value that is outside of the corresponding DDCRZ, and therefore, the characteristic NAA/Cho (PWMR) has $\bar{NC}=1$.


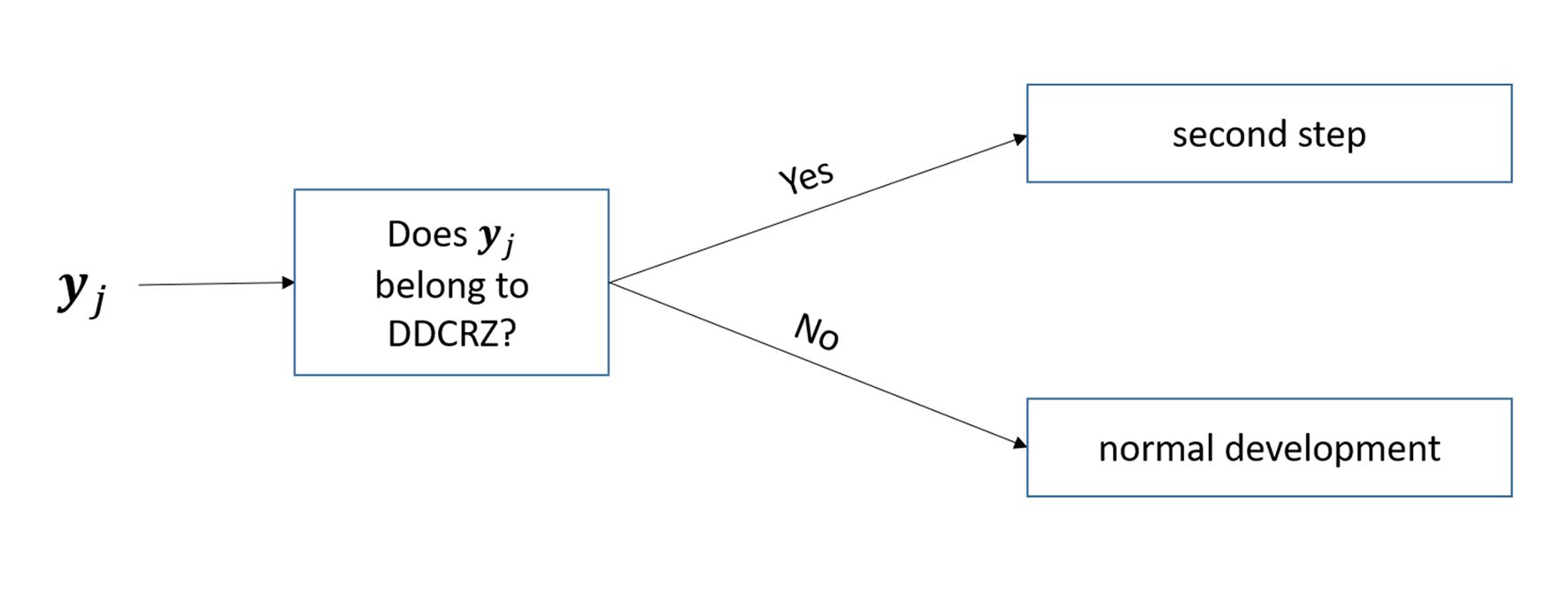


Figure 2. The illustration of the first step of the proposed predictor.


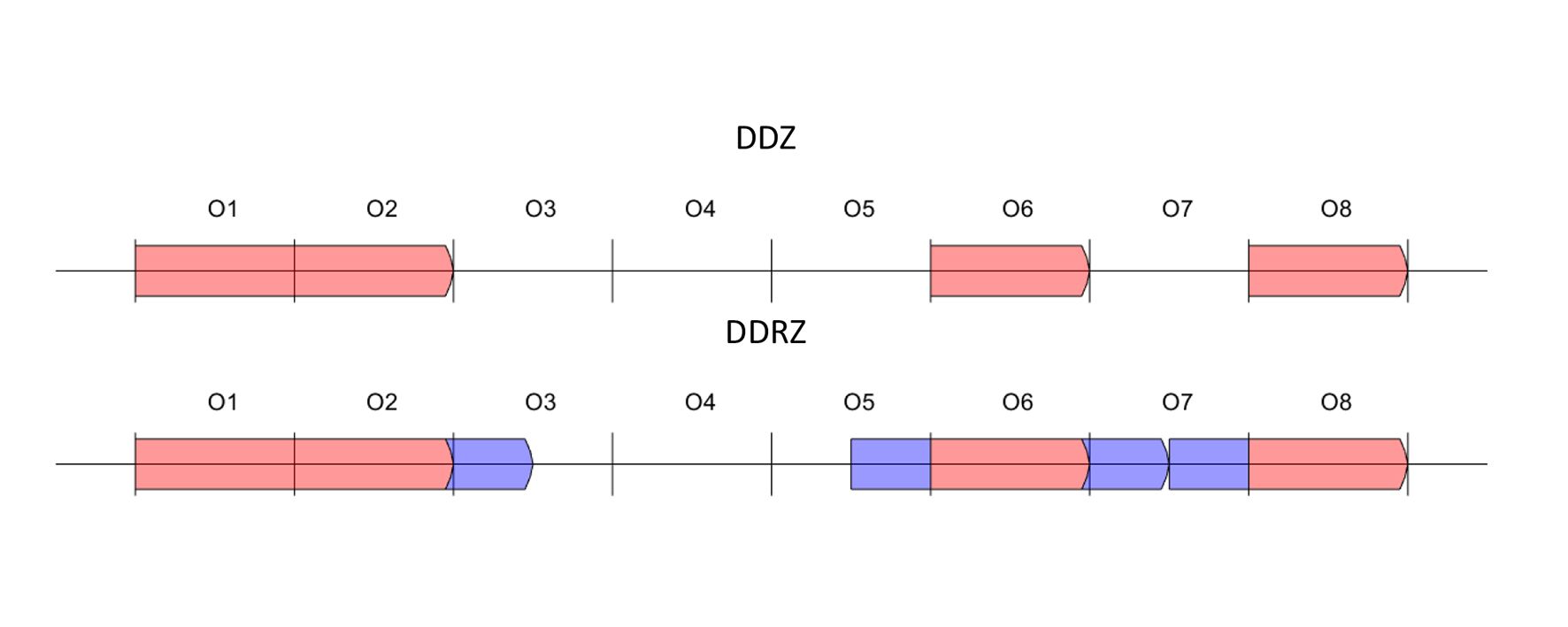


Figure 3. A schematic illustration of the creation of a developmental delay relaxed zone (DDRZ, lower image) from a developmental delay zone (DDZ, upper image).


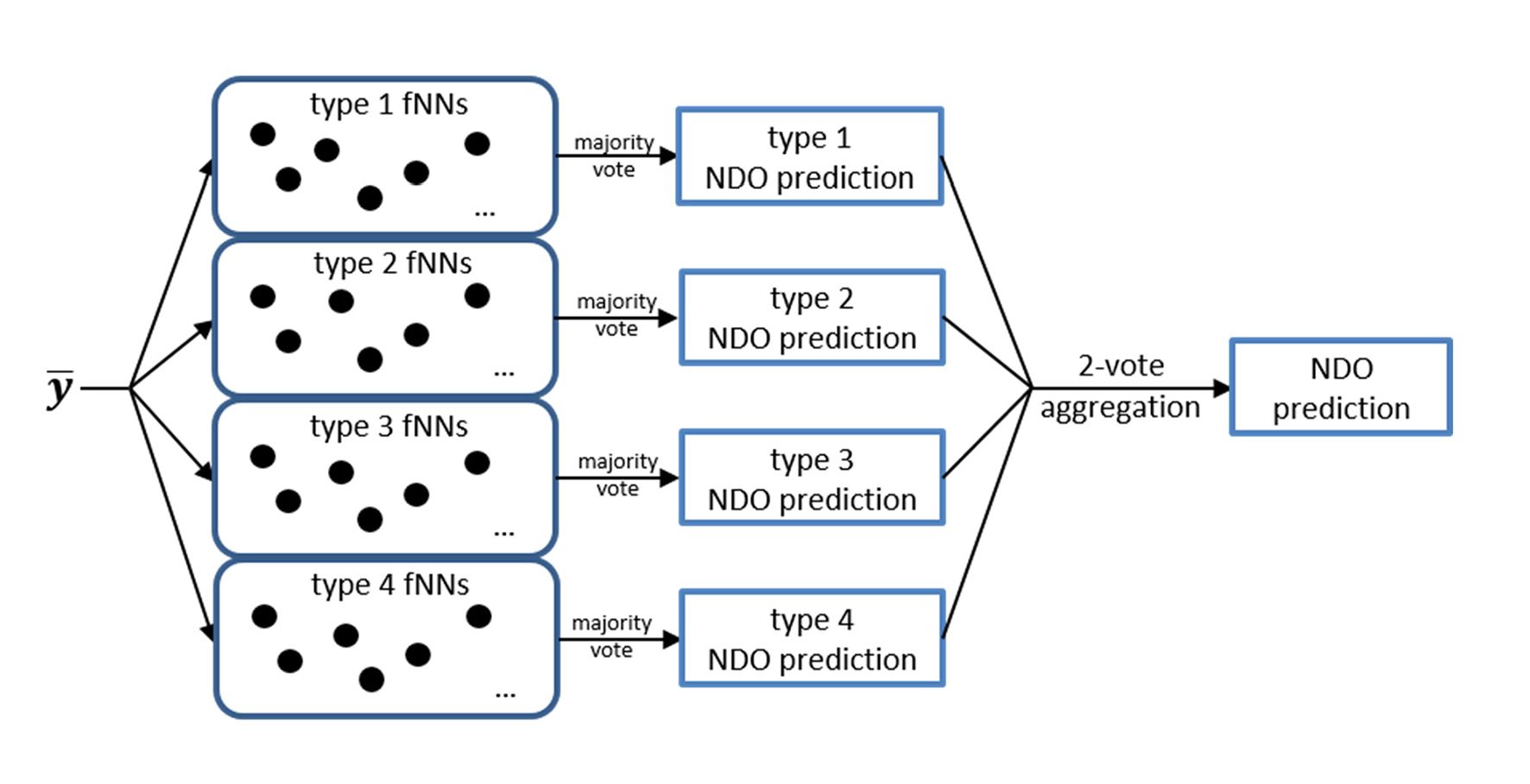


Figure 4. The illustration of the second step of the proposed predictor.

**Tables**


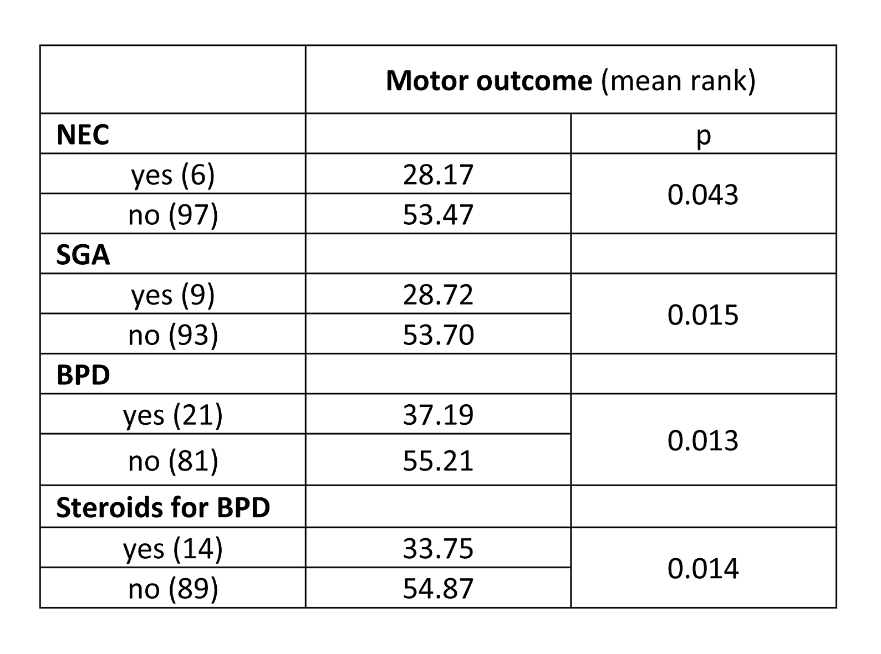


Table 1a. Group differences for the clinical data of the included preterm neonates.

Comment to Table 1a:

We found a significant difference in the motor outcome between the preterm neonates with and without necrotizing enterocolitis (NEC), bronchopulmonary dysplasia (BPD) and postnatal therapy for BPD as well as between the neonates small (SGA) and appropriate for gestational age. The impact of the clinical risk factors including BPD, NEC, and sepsis on the NDO in preterm infants has been reported previously [5-7]. Being SGA is significantly associated with BPD, but not with increased mortality [8]. Moreover, the impact of being SGA on the cognitive outcome at school age has been proved as well [9]. The effect of being SGA on motor outcome has not been reported previously. Since the number of the developmentally delayed preterm neonates is low, the consideration of the covariates would lead to the overparameterization of our models, which then would lead to the unrealistically high prediction accuracy of our models. As these results were “incidental findings” and we did not employ them as covariates, we are going to deeper analyze all these findings in a further study with more preterm neonates included.

**References**

1. Jerome F, Hastie T, Tibshirani R (2008). The elements of statistical learning. Second Edition. New York: Springer series in statistics.
2. Guyon I, Elisseeff A. An introduction to variable and feature selection. Journal of machine learning research. 2003;3(Mar):1157-82.
3. Sun Y, Wong AK, Kamel MS. Classification of imbalanced data: A review. International journal of pattern recognition and artificial intelligence. 2009 Jun;23(04):687-719.
4. Krawczyk B. Learning from imbalanced data: open challenges and future directions. Progress in Artificial Intelligence. 2016 Nov 1;5(4):221-32.
5. van Vliet EO, de Kieviet JF, Oosterlaan J, van Elburg RM. Perinatal infections and neurodevelopmental outcome in very preterm and very low-birth-weight infants: a meta-analysis. JAMA pediatrics. 2013 Jul 1;167(7):662-8.
6. Rose J, Vassar R, Cahill-Rowley K, et al. Neonatal physiological correlates of near-term brain development on MRI and DTI in very-low-birth-weight preterm infants. NeuroImage: Clinical. 2014 Jan 1; 5:169-77.
7. Neubauer V, Junker D, Griesmaier E, Schocke M, Kiechl-Kohlendorfer U. Bronchopulmonary dysplasia is associated with delayed structural brain maturation in preterm infants. Neonatology. 2015;107(3):179-84.
8. Nobile S, Marchionni P, Carnielli VP. Neonatal outcome of small for gestational age preterm infants. European journal of pediatrics. 2017 Aug 1;176(8):1083-8.
9. Guellec I, Lapillonne A, Renolleau S, et al., EPIPAGE Study Group. Neurologic outcomes at school age in very preterm infants born with severe or mild growth restriction. Pediatrics. 2011 Apr 1;127(4): e883-91.
